# Impaired endogenous fibrinolysis at high shear using a point-of-care test in STEMI is associated with alterations in clot architecture

**DOI:** 10.1007/s11239-018-01799-1

**Published:** 2019-02-09

**Authors:** Nikolaos Spinthakis, Ying Gue, Mohamed Farag, Guogang Ren, Manivannan Srinivasan, Anwar Baydoun, Diana A. Gorog

**Affiliations:** 1 Postgraduate Medical School, University of Hertfordshire, Hertfordshire, UK; 2 Department of Cardiology, East and North Hertfordshire NHS Trust, Hertfordshire, UK; 3 School of Engineering and Technology, University of Hertfordshire, Hertfordshire, UK; 4 National Heart & Lung Institute, Imperial College, Dovehouse Street, London, SW3 6LR UK

## Abstract

Impaired endogenous fibrinolysis is an adverse prognostic biomarker in acute coronary syndrome (ACS). Abnormally dense in vitro fibrin thrombi have been demonstrated in ACS patients and related to hypofibrinolysis using cumbersome, laboratory-based methods. We aimed to assess endogenous fibrinolysis using a point-of-care technique and relate this to clot architecture. From patients with ST-segment elevation myocardial infarction (STEMI), venous blood was drawn immediately on arrival to assess thrombotic status. Blood was assessed using the point-of-care Global Thrombosis Test which measures occlusive thrombus formation under high shear and subsequently endogenous fibrinolysis (lysis time, LT). Two samples per patient were run in parallel. In one channel, the measurement was allowed to proceed as normal. In the other, after occlusion, thrombus was extracted, washed, fixed in glutaraldehyde, dried, sputter-coated, and assessed using scanning electron microscope. Endogenous fibrinolysis was strongly associated fibrin fibre thickness (*p* = 0.0001). As LT increased (less efficient fibrinolysis), the fibrin network of the thrombus was significantly more compact and dense, with thinner fibrin fibres and smaller gaps. Fibrin fibre thickness correlated inversely with LT (r = − 0.89, *p* = 0.001). Adverse clot architecture in vitro is directly related to impaired endogenous fibrinolysis using a relatively new point-of-care technique in patients with STEMI. This may transform the relevance of fibrin clot architecture from an off-line laboratory association to being directly relevant to endogenous fibrinolysis at the patient bedside, which could be used as a near-patient test to guide prognosis and assess the effect of treatment.

## Highlights

- Abnormally dense in vitro fibrin thrombi have been associated with hypofibrinolysis using cumbersome laboratory-based methods.
- We show for the first time that, in STEMI patients, impaired endogenous fibrinolysis measured using a relatively new *point-of-care technique* is strongly related to adverse fibrin clot characteristics in vitro.
- This may transform the relevance of fibrin clot architecture from an off-line laboratory association to being directly relevant to endogenous fibrinolysis at the patient bedside.
- This could be used as a near-patient test to guide prognosis and assess the effect of treatment.

## Introduction

The likelihood of coronary thrombosis causing arterial occlusion in ST-elevation myocardial infarction (STEMI) depends on the effectiveness of endogenous fibrinolysis [1]. Impaired fibrinolysis is a recently-described adverse prognostic biomarker in acute coronary syndrome [2–4]. Abnormally dense fibrin thrombi have been demonstrated in populations at risk of coronary thrombosis [5] and related to hypofibrinolysis using cumbersome, laboratory-based, low-shear turbidimetric methods. We aimed to assess endogenous fibrinolysis under high shear using a near-patient technique and relate this to clot architecture.

## Methods

This prospective observational study was approved by National Research Ethics Service and the UK Health Research Authority (ClinicalTrials.gov identifier: NCT02562690). Following consent, 50 patients presenting with STEMI with a view to primary percutaneous coronary intervention (PPCI) were screened to assess thrombotic status, to identify 15 patients who were included. Dual antiplatelet therapy was administered in the ambulance pre-arrival. Patients with coagulopathy, malignancy, sepsis, blood dyscrasias (platelets < 100 × 10^9^/L, haemoglobin < 80 g/L) or taking anticoagulants were excluded.

### Assessment of endogenous fibrinolysis

Venous blood drawn upon arrival, before heparin or PPCI, was immediately assessed using the point-of-care Global Thrombosis Test (GTT, Thromboquest Ltd., UK) situated in the catheterization laboratory. This measures the time taken for occlusive thrombus formation under high shear (occlusion time, OT) and subsequently the time until restart of flow due to endogenous fibrinolysis (lysis time, LT) [3, 4]. Two samples per patient were run in parallel. In one channel, the measurement was allowed to proceed as normal. In the other, immediately after occlusion, the thrombus was extracted and fixed.

### Scanning electron microscopy

The thrombus was washed in Na-cacodylate buffer, fixed in 2.5% glutaraldehyde, critically dried with alcohol (5–100%) followed by hexamethyldisilazane, sputter-coated with gold with Quorum 150 [6], and imaged using scanning electron microscope (SEM) (Lambda Photometrics Ltd., UK). Thickness of fibrin fibres (n = 100) from nine areas per thrombus were measured. Fibrin density was calculated using a Scentis Database Image software with a Multiple Phase Percentage Processing package, based on differential light intensities, to derive total fibrin fibre area (% per visual field [p.v.f.]) indicating fibrin density and void space-gap (% p.v.f.), an indirect measure of clot density. SEM was performed blinded to thrombotic status results.

### Statistical analysis

Patients were divided into quartiles based on LT. Kruskal–Wallis test was used to assess differences between groups, Wilcoxon signed-rank test to investigate differences in different areas within clots and correlation assessed with Spearman’s method. Analyses were performed with Stata V15.1 (StataCorp, USA).

## Results

**Fig. 1:**
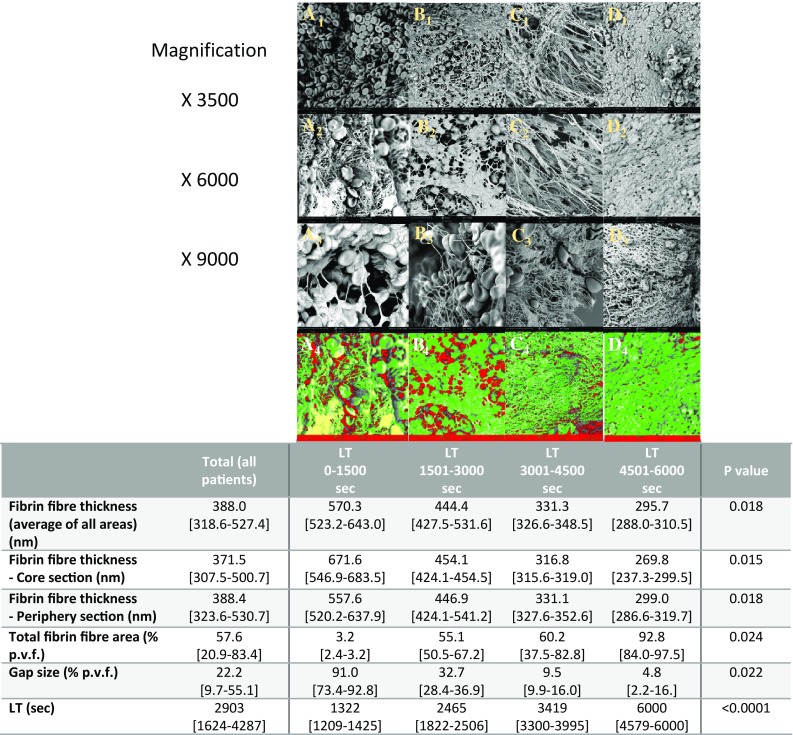
Representative SEM images of in vitro thrombus from patients with increasing fibrinolysis time (panels **a** → **d**), at increasing magnification (1 → 3), and differential light intensities (**a**4 → **d**4). Images relate directly to LT quartiles in table of clot characteristics (median [interquartile range]) below

Endogenous fibrinolysis was strongly associated fibrin fibre thickness (*p* = 0.0001). As LT increased (less efficient fibrinolysis), the fibrin network of the thrombus was significantly more compact and dense, with thinner fibrin fibres and smaller gaps (Fig. 1). Fibrin fibre thickness correlated inversely with LT (r = − 0.89, *p* = 0.001) but not with OT (*p* = 0.688).

Clinical characteristics, hemoglobin, hematocrit, platelet count, fibrinogen, troponin and pain-to-door time were not related to LT or fibrin thickness. After accounting for clinical parameters known to impact fibrinolysis (age, hypertension, diabetes, haematological and biochemical characteristics), the relationship between LT and fibrin fibre thickness remained significant (*p* = 0.005) on multivariable regression.

## Conclusion

Patients with STEMI who exhibit impaired endogenous fibrinolysis under high shear using a point-of-care technique, create in vitro thrombi with much denser fibrin meshwork composed of thinner fibrin fibres and smaller gaps, than patients with effective fibrinolysis. This is the first study correlating a point-of-care measure of endogenous fibrinolysis with in vitro fibrin clot characteristics. Previous studies have shown unfavourable clot characteristics in high-risk groups, using cumbersome, off-line, turbidimetric laboratory methods employing low shear. This new evidence base linking thrombus architecture to cardiovascular risk could now be translated from an off-line laboratory association to a clinically meaningful, near-patient assessment of endogenous fibrinolysis, with the potential to modify risk.
